# Total, Soluble and Insoluble Oxalate Contents of Ripe Green and Golden Kiwifruit

**DOI:** 10.3390/foods2010076

**Published:** 2013-03-05

**Authors:** Hà Vũ Hồng Nguyễn, Geoffrey P. Savage

**Affiliations:** 1 Food Technology Department, Biotechnology School, International University, Ho Chi Minh City, 70000, Vietnam; E-Mail: nvhha@hcmiu.edu.vn; 2 Food Group, Department of Wine, Food and Molecular Biosciences, Agriculture and Life Sciences, Lincoln University, Lincoln 7647, Christchurch, New Zealand

**Keywords:** green kiwifruit (*Actinidia deliciosa *L.), golden kiwifruit (*Actinidia chinensis *L*.*), total, soluble and insoluble oxalates

## Abstract

Three bulk samples of two different cultivars of kiwifruit, green (*Actinidia deliciosa *L*.*) and golden (*Actinidia chinensis *L*.*) were bought ripe, ready to eat from a local market. The aim of the study was to determine the oxalate composition of each of the three fractions of kiwifruit, namely skin, pulp and seeds. The pulp consisted of 90.4% of the edible portion of the two cultivars while the skin and seeds made up a mean of 8.0% and 1.6% respectively. Total oxalate was extracted with 2.0 M HCL at 21 °C for 15 min and soluble oxalates extracted at 21 °C in water for 15 min from each fraction. The total and soluble oxalate compositions of each fraction were determined using ion exchange HPLC chromatography. The pulp of golden kiwifruit contained lower amounts of total oxalates (15.7 *vs.* 19.3 mg/100 g FW) and higher amounts of soluble oxalates (8.5 *vs.* 7.6 mg/100 g FW) when compared to the green cultivar. The skin of the green cultivar contained lower levels of insoluble oxalates (36.9 *vs.* 43.6 mg/100 g FW), while the seeds of the green cultivar contained higher levels of insoluble oxalates 106.7 *vs.* 84.7 mg/100 g FW.

## 1. Introduction

Kiwifruit is currently one of New Zealand’s largest horticultural export crops with 380,000 tonnes being harvested in 2009 and 361,066 tones being exported to over 55 countries in the same year [1]. Kiwifruit has also become a significant fruit in the New Zealand domestic market. Early studies have shown that green kiwifruit contained moderate amounts of total oxalate which is largely found as calcium oxalate in raphide crystals. These crystals of insoluble oxalate are responsible for the irritant factor or “catch” which is used to describe an irritation of the mucous membranes of the mouth [2]. Soluble oxalates, however, may be of interest for kidney stone patients who are trying to decrease their urinary oxalate excretion by avoiding the consumption of oxalate-rich foods [2,3,4]. The amounts of total oxalates in golden kiwifruit reported in the literature varies from 7.8 to 45 mg/100 g fresh weight (FW), while the values for green kiwifruit ranged from 12.7 to 84.3 mg/100 g FW [2,4,5,6]. 

While many authors report soluble and insoluble oxalate as separately measurable components of the oxalate content of foods [2,6,7,8,9]. Soluble oxalate was not detected in six different genotypes accessed from China [10]. The total oxalate content of these genotypes measured using HPLC ranged from 18–45 mg/100 g FW. In these studies, very small volumes were used to extract total and soluble oxalates and fruit acids had a considerable effect on the pH of the water extraction medium. In the majority of studies, larger volumes of water are used to extract soluble oxalates and plant acids have little effect on the pH of the extraction medium even though it is an un-buffered medium. Recent studies have shown that the pH of the original deionized water was 6.9 which changed to an average of 6.0 for samples of green and golden kiwifruit at the end of the 15 min extractions [11]. This would have a minimal effect on the extraction of soluble oxalates from the kiwifruit tissue. The concentration of Ca^2+^ and the extraction temperature could also have an effect on efficiency of oxalate extraction from the tissue. In a recent study, the extraction of soluble oxalate from Zespri green kiwifruit at 80 °C was increased by 1.5% (6.5 *vs.* 6.6 mg/100 g FW) compared to the extraction at room temperature. In contrast, the extraction of total oxalate was increased by 22.2% (from 30.6 *vs.* 37.4 mg/100 g FW) [11]. 

Although the marked differences in the oxalate composition of kiwifruit may be due to genotype [10] growing conditions [6] and harvest maturity stage [12], using unreliable analytical methods could also result in an under- or over-estimation of oxalate contents [11].

Commercially-grown kiwifruit are harvested when physiologically mature and can be stored for several months [13], while locally grown fruit is picked at an “eating ripeness” stage when its optimum nutrition and sensory features have been reached. So far, the oxalate content of New Zealand kiwifruit has only been reported in a few publications [10,12] which reported total oxalate, but not soluble oxalate content of kiwifruit. In contrast, an earlier study of six different cultivars [2] showed that the total oxalate content of six different New Zealand grown ranged from 37.0 to 65.2 mg/100 g FW and 15.8 to 41.4 mg soluble oxalate/100 g FW. The objective of this study was to determine the total, soluble and insoluble oxalate contents in different fractions of green and golden kiwifruit cultivars as it is clear that many earlier studies have measured the oxalate content of whole kiwifruit and overlooked the fact that the skin is not often consumed. 

## 2. Experimental Section

### 2.1. Sample Preparation

Three separate bulk samples of green kiwifruit (*Actinidia deliciosa* cv. Hayward) and golden kiwifruit (*Actinidia chinensis* cv. Hort16A) were bought fresh from Growers Direct Market Ltd., Christchurch, New Zealand. A characteristic feature of green kiwifruit is that the skin is covered with short green hairs and this means that the skin is not commonly eaten, in contrast, golden kiwifruit has a smooth skin and these fruits can be eaten whole. A feature of both fruits is that the skin and the pulp are light green and golden in colour. Fruits from each of the three bulk samples weighing between 80 and 100 g were carefully inspected; overripe and damaged fruits were excluded. Five fruits were randomly selected from each of the three bulk samples and they were divided into three fractions: skin, pulp and seeds using a stainless-steel knife. The pooled fractions were weighed and expressed as a percentage of the total weight of the fruit. All samples were immediately frozen with liquid N_2_ and then finely ground using a mortar and pestle. 

### 2.2. Moisture Content

The moisture content of each fresh fraction of kiwifruit was determined in triplicate by drying in an oven at 105 °C until a constant weight was achieved [14].

### 2.3. Extraction and Determination of Total and Soluble Oxalate

Extraction of total and soluble oxalates was carried out in triplicate using the method of Savage *et al.* [9]. Total oxalates were extracted from 1.0 g samples of each freeze-dried powdered fruit fraction with 40 mL 2.0 M HCL at 21 °C for 15 min and soluble oxalates were extracted from each 1.0 g samples of freeze-dried powdered fruit with 40 mL of nanopure water (Barnstead International, Dubuque, IA, USA) at 21 °C for 15 min. The extracts were allowed to cool and then transferred quantitatively into 100 mL volumetric flasks and made up to volume. The extracts were centrifuged at 2889 rcf for 15 min. The supernatant was filtered through a 0.45 µm cellulose nitrate filter (Sartorious, Göttingen, Germany). The chromatographic separation was carried out using a 300 × 7.8 mm Rezex ROA ion exclusion organic acid column (Phenomenex, Torrance, CA, USA) attached to a cation H+ guard column (BioRad, Richmond, CA, USA). The analytical columnwas held at 25 °C. The equipment consisted of an auto sampler (Hitachi AS-2000, Hitachi Ltd., Kyoto, Japan), a ternary Spectra-Physics, SP 8800 HPLC pump (Spectra-Physics, San Jose, CA, USA), a Waters, U6K injector (Waters Inc., Marlborough, MA, USA), a UV/VIS detector Spectra-Physics SP8450 (Spectra-Physics, San Jose, CA, USA) set on 210 nm. Data capture and processing were carried out using a peak simple chromatography data system (SSI Scientific Systems Inc, State College, PA, USA). The mobile phase used was an aqueous solution of 25 mM sulphuric acid. Samples (20 µL) were injected onto the column and eluted at a flow rate of 0.6 mL/min. The oxalic acid peak was identified by comparison of the retention time to a range of common plant organic acid standards.

### 2.4. Standard Calibration

Two standard curves were prepared in the range 1–20 mg/100 mL as all samples came within this linear range; one set of standard solutions were prepared by adding oxalic acid (99.999% pure, Sigma-Aldrich Co., St Louis, MO, USA) to 100 mL volumetric flasks and making up to volume with distilled water. This set was used to analyse the soluble oxalic acid content of the water extracts. A further set of standard solutions were prepared by diluting the standard oxalic acid to 100 mL with 2.0 M HCI. This set of standards was used to quantify the total oxalic acid content of the samples. All blank and standard solutions were filtered through a 0.45 mm cellulose acetate membrane syringe filter prior to analysis. The limit of detection of oxalic acid was 0.01 mg oxalate/100 mL in both water and acid extraction methods.

### 2.5. Recovery Study

The recovery of oxalic acid during extraction was determined by adding 10 mg of oxalic acid (Sigma-Aldrich Co., St Louis, MO, USA) to 1 g freeze-dried kiwifruit powder. These samples were subsequently extracted using deionised water (for soluble oxalates) and 2 M HCL (for total oxalates) and analysed in triplicate using the method as outlined above.The mean recoveries of added oxalate using 2 M HCL were 98.5% ± 1.6%, while the mean recovery using deionised water extraction was 96.3% ± 1.9%. 

The insoluble oxalate content was calculated as the difference between the total oxalate (acid extract) and soluble oxalate (water extract) [15]. Each sample was analysed in triplicate and the results are presented as mean mg oxalate/100 g FW ± standard error (SE).

### 2.6. Statistical Analysis

The results are presented as mean of three determinations ± standard error. Statistical analysis using two-way analysis of variance (cultivar × fraction) was performed using GenStat version 13 for Windows 7 (VSN International Ltd., Hemel Hempstead, Hertfordshire, UK) to measure the differences between the oxalate content of the skin, pulp and seeds of the green and golden cultivars of kiwifruit. 

## 3. Results and Discussion

When purchased, the two cultivars of kiwifruit had reached an acceptable stage of ripeness for immediate consumption. The distribution of the skin and pulp of each cultivar were similar, except that the green kiwifruit cultivar contained a higher proportion of seeds compared to golden kiwifruit of similar size and weight (Table 1). The dry matter of the golden kiwifruit skin, which was smooth, was much higher than the hairy green kiwifruit skin. The total, soluble and insoluble oxalate contents of the skin, pulp and seeds for green and golden kiwifruit are shown in Table 2. In both cultivars, the amounts of total oxalates found in the seeds were highest, followed by the skin and then in the pulp. Total oxalate contents of the pulp and the seeds of green kiwifruit were significantly higher than those found in golden kiwifruit. In contrast, the skin of green kiwifruit contained lower total oxalates compared to the golden cultivar. This is important as the pulp makes up the major portion of the fruit that is eaten, as the skin of the green cultivar is usually not eaten. 

**Table 1:** The % distribution and dry matter (g/100 g FW) of the three fractions of ripe, ready to eat green and gold kiwifruit (*n* = 3).

**Fraction of the fresh fruit**		**% Distribution**		**Dry matter**	
		Green	Golden	Green	Golden
Skin		7.3 ± 0.1	8.6 ± 0.4	28.1 ± 0.6	38.1 ± 0.2
Pulp		90.3 ± 0.1	90.6 ± 0.4	14.4 ± 0.1	15.2 ± 0.3
Seeds		2.2 ± 0.1	0.9 ± 0.1	82.6 ± 1.1	81.3 ± 2.2
		**Significance**			
**Analysis of variance**	**df**	**% Distribution**		**Dry matter**	
Fraction	2	NS		***	
Cultivar	1	***		***	
Fraction * cultivar	2	***		***	
l.s.d (5%) within cultivar		0.4		1.7	
l.s.d (5%) within fraction		0.5		2.1	
l.s.d (5%) between cultivar		0.7		3	

NS: not significant; * p< 0.05; ** p < 0.01; *** p < 0.001; l.s.d: least significant difference.

**Table 2:** Mean total, soluble and insoluble oxalate contents (mg/100 g FW ± SE) of the skin, pulp and seed fractions of ripe, ready to eat green and golden kiwifruit, the values in parenthesis are the ratio of soluble to total oxalate for each cultivar (*n* = 3).

	**Total oxalate**			**Soluble oxalate**			**Insoluble oxalate**		
	Green	Golden		Green		Golden	Green		Golden
Skin	47.7 ± 0.2	55.4 ± 1.1		10.8 ± 0.2 (22.6)		11.9 ± 0.3 (21.4)	36.9 ± 0.2		43.6 ± 1.1
Pulp	19.3 ± 0.3	15.7 ± 0.1		7.6 ± 0.2 (39.4)		8.5 ± 0.1 (54.1)	11.7 ± 0.3		7.2 ± 0.1
Seeds	116.9 ± 2.8	97.3 ± 1.4		10.2 ± 0.2 (8.7)		12.6 ± 0.3 (12.9)	106.7 ± 2.7		84.7 ± 1.4
			**Significance**						
**Analysis of variance**		**df**	**Total oxalate**		**Soluble oxalate**			**Insoluble oxalate**	
Fraction		2	***		**			***	
Cultivar		1	***		**			***	
Fraction * cultivar		2	***		***			***	
l.s.d (5%) within cultivar			2.4		0.4			2.5	
l.s.d (5%) within fraction			2.9		0.5			3.0	
l.s.d (5%) between cultivar			4.2		0.7			4.3	

NS: not significant; * p< 0.05; ** p < 0.01; *** p < 0.001; l.s.d: least significant difference.

The soluble oxalate content of the two cultivars was similar with the golden cultivar having marginally higher values. Except for the skin of golden kiwifruit, which contained significantly (*p* < 0.001) higher levels of insoluble, the oxalate levels amounts inthe pulp and seeds were higher in the green kiwifruit cultivar.

On a fresh weight basis, the total oxalate content in the skin of the golden kiwifruit was 55.4 mg/100 g FW in comparison with 20–60 mg/100 mg FW reported for six genotypes of golden kiwifruit [10]. Using microscopic and digital camera observation, it was shown that there was a higher accumulation of oxalate crystals in the inner-pericarp area, the region defined as the tissues adjacent to the seed [8,12]. It was difficult to compare the total oxalate level of the pulp in this study with the values obtained earlier [10] because the pulp in the present study included the outer-pericarp, inner-pericarp and core but not the skin and seeds. In this study, the skin of both cultivars contained high levels of insoluble oxalate and low levels of soluble oxalates. The higher insoluble oxalate levels in the skin than in the pulp may represent a functional mechanism to protect fruit from insects and herbivores [10]. It was found in this study that depending on the cultivar, the distribution of oxalates in the different fractions was different between the cultivars. This finding supported the results obtained earlier [12]. Soluble oxalate contents of the seeds were similar to the amounts found in the skin. The oxalates are concentrated in the outer cell layers such as the coat or testa of the seeds in the form of small crystals [10]. Overall the oxalate content of green and gold kiwifruit are low compared to the levels found in some New Zealand fruit and very low compared to selected imported tropical fruit [4]. Regular consumption of green and gold kiwifruit would not significantly increase the daily intake of oxalates in the diet and therefore would not pose any risk to people who are at risk of forming kidney stones.

## 4. Conclusions

Overall, golden kiwifruit contained significantly lower (*p* < 0.001) levels of total oxalates in the pulp and seeds, while the green kiwifruit cultivar contained marginally lower (*p* < 0.01) soluble oxalates in these two fractions while the skin of the golden cultivar contained higher levels of total oxalate. In contrast, the soluble oxalate contents of each of the three fractions of the golden cultivar were marginally higher than the green cultivar. This study confirms that the oxalate contents of ready to eat green and gold kiwifruit are low and unlikely to significantly increase the daily intake of oxalates in the diet.
